# Evaluation of CYP2C9 Activity in Rats: Use of Tolbutamide Alone and in Combined with Bupropion

**Published:** 2014

**Authors:** Xiangjun Qiu, Jijun Song, Hongchang Yuan, Yi Hou, Xiaofeng Pan, Ren-ai Xu

**Affiliations:** a *Medical College of Henan University of Science and Technology, Luoyang 471003,China. *; b *Children’s Hospital of Zhengzhou, Zhengzhou 450053, China.*; c *The First Affiliated Hospital of Wenzhou Medical University, Wenzhou 325000, China.*

**Keywords:** Tolbutamide, Bupropion, P450, Probe drug, Cocktail, Interaction

## Abstract

A “cocktail”of several probe drugs is often used to evaluate metabolic activity of multiple cytochrome P450 enzymes in one session. Some interactions among probe drugs can appear and may impact the rate of biotransformation of other ones. Our presented work was aimed on the influence of bupropion on cytochrome P450-mediated metabolism of tolbutamide. The biotransformation rates of tolbutamide administered either separately or in combined with bupropion were compared in this study. The results revealed that bupropion had no significant effect on tolbutamide hydroxylation. Thus, due to stability in cytochrome P450 enzyme metabolic activity in the case of combining of two model probe drugs the procedure can show to no extent differential results comparing to the single-marker use.

## Introduction

Specific probe drugs have been widely used for assessing various individual cytochrome P450 (CYP) enzymes activities. In addition to phenotying, this approach can be used to evaluate the potential inhibitory or abductive effects of a New Chemical Entity (NCE) on the pharmacokinetics of representative probes of CYP enzymes and hence, in combination with *in-vitro* data, provide the basis for a rational and efficient approach to a drug-drug interaction strategy. Therefore, a number of drug metabolism cocktail methods have been described and developed (1-7). 

Compared to the administration of individual specific probes in multiple studies, two well-known advantages of this so-called “cocktail” approach are assessing the metabolic activity of multiple CYP in a single experiment (8) and minimizing the inter- and intra-subject variability (9), respectively. Unfortunately, the disadvantages of this approach also has certain limitations, such as mutual interactions between probe drugs, the frequent occurrence of side effects, and analytical complexities (8). For example, interactions between chlorzoxazone (CYP2E1) and midazolam (CYP3A) (10), dextromethorphan (CYP2D6) and chloroguanide (CYP2C19) (11), and caffeine (CYP1A2) and chlorzoxazone(CYP2E1) (12) have been reported. Thus, it is essential to ensure that probe drugs and metabolites in a serum or urine sample do not give rise to analytical interference.

Our current work was to determine whether the pharmacokinetic parameters of tolbutamide (TB) and its metabolite hydroxytolbutamide (HTB), and the metabolic ratios in urine were influenced by administration of TB alone and in combined with bupropion (BUP) in an oral cocktail intake. 

## Experimental

*Materials*

TB(purity > 98.0%), HTB(purity > 98.0%) and BUP(purity > 98.0%) were purchased from Sigma-Aldrich Company. Twelve male Sprague-Dawley rats, weighing between 200-250 g, were all obtained from Wenzhou Medical College Laboratory Animal Center (Wenzhou, China). The rats were housed individually in standard plastic cages and maintained on normal rat purina chow and fresh water ad libitum in a room controlled for temperature (23-25 ^o^C) and lighting (8:00-20:00 light, 20:00-8:00 dark). After the 1 week acclimatization period, the rats were used for experiments and all efforts were made to minimize any animal suffering. All experimental procedures and protocols were reviewed and approved by the Animal Care and Use Committee of Wenzhou Medical College and were in accordance with the Guide for the Care and Use of Laboratory Animals. 

*HTB/TB urinary metabolic ratio* *study*

This was an open, randomized crossover study. Twelve male Sprague-Dawley rats were randomly divided into two groups (n=6), respectively. Before the study, diet was prohibited for 12 h until 2 h after drug administration, but water was freely available. In the first cycle, one group took TB(3 mg/Kg) alone and another group received TB(3 mg/Kg) and BUP(15 mg/Kg) in combination after an overnight fast. Drugs were administered by gastric irrigation. Urine was collected during time intervals of 0 to 8 hours, 8 to 12 hours, and 12 to 24 hours after drug intake. Before determination, each urine sample was diluted appropriate concentration.

*Pharmacokinetic* *s* *tudy*

The above 12 rats were raised for a 2-week recovery stage for the secondary administration. In the secondary cycle, oral administration in the first cycle were repeated. Blood samples (0.3 mL) from the tail vein were collected immediately into heparinized ploythene tubes before drug administration and 0.083, 0.25, 0.5, 0.75, 1, 1.5, 2, 3, 4, 5, 6, 8, 12, 24, 36 and 48 h thereafter. After the samples centrifuged at 13,000 rpm for 10 min, the plasma obtained (100 μL) was transferred into 1.5 mL heparinized ploythene tubes and stored at -20 ^o^C until analysis. 

*LC/MS analysis of TB and its metabolite* *HTB*

For the analysis of TB and its metabolite HTB, a 1200 Series liquid chromatograph (Agilent Technologies, Waldbronn, Germany) equipped with a quaternary pump, a degasser, an autosampler, a thermostatted column compartment, and a Bruker Esquire HCT mass spectrometer (Bruker Technologies, Bremen, Germany) equipped with an electrospray ion source were used. Chromatographic separation was performed on a Agilent Zorbax SB-C18 column (150 mm × 2.1 mm, 3.5 μm particle) at 30 °C by using the gradient elution of 0.1% formic acid in water (mobile phase A) and acetonitrile (mobile phase B) as follows: 0-1.5 min (10-85% B), 1.5–6.0 min (85–85% B), 6.0–7.0 min (85-10% B), 7.0–10.0 min (10–10% B). The flow rate was 0.4 mL/min.

The quantification was performed by the peak-area method. Drying gas flow and nebuliser pressure was set at 6 L min^-1^ and 20 psi. Dry gas temperature and capillary voltage of the system were adjusted at 350 ^o^C and 3,500 V, respectively. LC-MS was performed with SIM mode using target ions at m/z 271.2 for TB and m/z 286.9 for HTB in positive ion electrospray ionization interface. The lower limits of quantification (LLOQ) for TB and HTB were 5 ng/mL and 1 ng/mL, respectively. The accuracy (% bias) and precision(% CV) of the analytical method were less than 5% and 8% for both analytes at all concentrations.

*Statistical analysis*

The results were reported as a mean ± SD. All analyses for comparing the datas determined from TB alone and in combination were performed with the SPSS software system version 16.0 (SPSS Inc., Chicago) by use of Student's t-test. The level of significance was set at P < 0.05. 

## Results and Discussion

**Figure 1:**
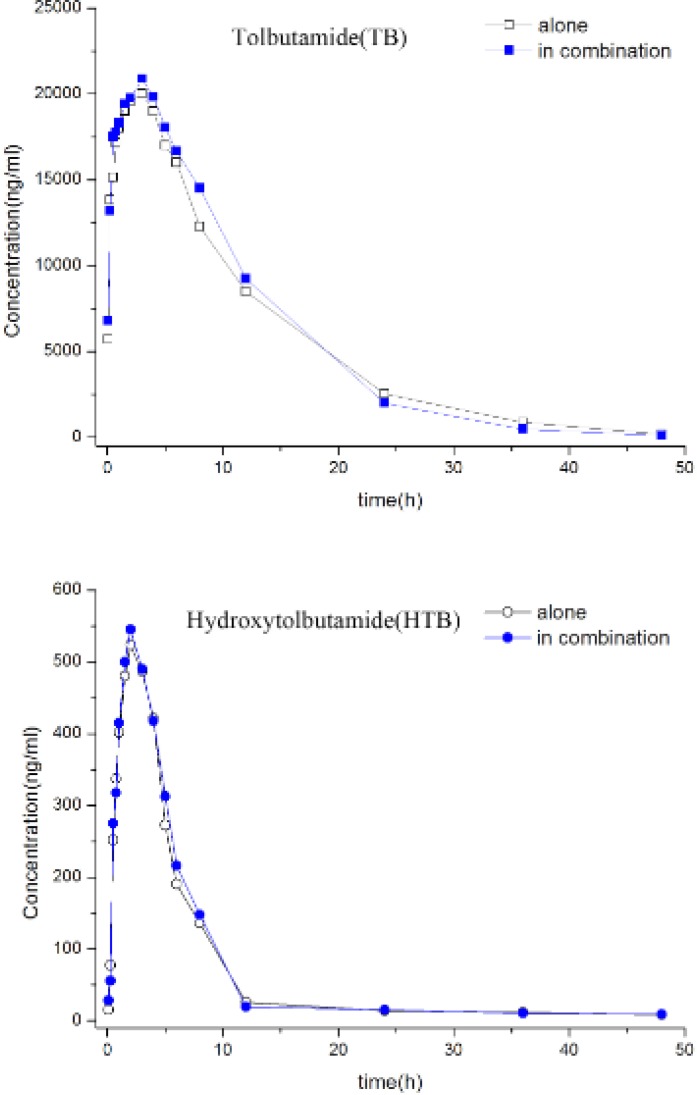
Median plasma TB and HTB concentrations following TB given alone and in combined with BUP versus time profile

Table 1 shows that TB partial metabolic clearance to HTB and renal clearance of TB and HTB did not change significantly whether TB was administered alone or in the presence of BUP. Moreover, the HTB/TB urinary metabolic ratio was not affect over a 24 h collection after simultaneous BUP oral administration. Likewise, the individual and mean TB and HTB plasma concentration-time profiles(Figure 1) were not significantly different whether TB was administered alone or with BUP. Accordingly, there was no difference in the pharmacokinetic parameters for TB and HTB and for the plasma HTB/TB metabolic ratio in the presence or absence of BUP. The results suggest that BUP addition to TB had no significant effect on CYP2C9-mediated TB hydroxylation.

**Table 1:** Urinary metabolic ratio of HTB/TB over 24 hours after administration of TB alone or in the presence of BUP

**Time** **(h)**	**TB/HTB urinary metabolic ratio**		**p-v** **alue**
	**TB alone**	**TB + BUP**	
0-8	55.86 ± 16.78	53.76 ± 15.89	NS
8-12	73.03 ± 19.57	74.23 ± 20.32	NS
12-24	78.73 ± 28.60	79.67 ± 25.10	NS

Values are expressed as mean ± SD. NS: Not significant.

The activity of CYP is most often evaluated using specific probe drug of distinct CYP enzyme. In addition, there are often used more probe drugs together, so as the activity of multiple CYP enzymes could be determined simultaneously and the latter is the basis of many clinical studies in the field of drug metabolism and pharmacogenetics. Previous studies have already shown that TB is an appropriate probe to assess CYP2C9 activity in humans (7,13). For practical reasons, cocktail approachs including TB and BUP were established to assess the activities of CYP2C9, CYP2B6 and other CYP isoforms to study whether other chemicals or drugs may induce or inhibit CYP system and predict the potential drug-drug interactions (-).

Genetic determinants, dietary components, environmental factors, and medications can induce or inhibit enzyme activity. The possibility of pharmacokinetic and pharmacodynamics interactions between phentoyping probes requires that potential combinations be assessed before widespread use. Therefore, our work was aimed on the influence of BUP on CYP-mediated metabolism of TB. In our study, we observed that the plasma concentrations of TB and its metabolite HTB and the HTB/TB urinary metabolic ratio were not significantly changed whether TB was administered alone or in the presence of BUP. This result indicated that measurement of CYP2C9 activity with use of TB as a probe was not altered by coadministration of BUP. Several conclusions of this phenomenon can be confirmed as follow: (1) TB was not influenced by the potential interference from the simultaneously administered BUP and their metabolites; (2) selectivity and sensitivity of TB was maintained and inhibition of BUP did not occur; (3) pharmacokinetic and pharmacodynamics interactions between TB and BUP was hardly little even not. 

Combination of the markers of metabolic activity in evaluating the CYP enzymes activity is quite often in practice. From the presented results, it may be suggested that BUP had no significant effect on TB hydroxylation.
